# Targeting HSP90 Inhibits Proliferation and Induces Apoptosis Through AKT1/ERK Pathway in Lung Cancer

**DOI:** 10.3389/fphar.2021.724192

**Published:** 2022-01-14

**Authors:** Mengyuan Niu, Bin Zhang, Li Li, Zhonglan Su, Wenyuan Pu, Chen Zhao, Lulu Wei, Panpan Lian, Renwei Lu, Ranran Wang, Junaid Wazir, Qian Gao, Shiyu Song, Hongwei Wang

**Affiliations:** ^1^ State Key Laboratory of Analytical Chemistry for Life Science, Medical School of Nanjing University, Nanjing, China; ^2^ Center for Translational Medicine and Jiangsu Key Laboratory of Molecular Medicine, Medical School of Nanjing University, Nanjing, China; ^3^ Wenzhou Medical University, Wenzhou, China; ^4^ Central Laboratory, Nanjing Chest Hospital, Affiliated Nanjing Brain Hospital, Nanjing Medical University, Nanjing, China; ^5^ Department of Dermatology, The First Affiliated Hospital of Nanjing Medical University, Nanjing, China

**Keywords:** Hsp90, 17-DMAG, lung cancer, AKT1, ERK

## Abstract

Lung cancer is one of the most common malignant cancers worldwide. Searching for specific cancer targets and developing efficient therapies with lower toxicity is urgently needed. HPS90 is a key chaperon protein that has multiple client proteins involved in the development of cancer. In this study, we investigated the transcriptional levels of HSP90 isoforms in cancerous and normal tissues of lung cancer patients in multiple datasets. The higher expression of HSP90AA1 in cancer tissues correlated with poorer overall survival was observed. The higher levels of transcription and expression of HSP90AA1 and the activity of AKT1/ERK pathways were confirmed in lung cancer patient tissues. In both human and mouse lung cancer cell lines, knocking down HSP90AA1 promoted cell apoptosis through the inhibition of the pro-survival effect of AKT1 by decreasing the phosphorylation of itself and its downstream factors of mTOR and BAD, as well as downregulating Mcl1, Bcl-xl, and Survivin. The knockdown also suppressed lung cancer cell proliferation by inhibiting ERK activation and downregulating CyclinD1 expression. The treatment of 17-DMAG, an HSP90 inhibitor, recaptured these effects *in vitro* and inhibited tumor cell growth, and induced apoptosis without obvious side effects in lung tumor xenograft mouse models. This study suggests that targeting HSP90 by 17-DMAG could be a potential therapy for the treatment of lung cancer.

## Introduction

Lung cancer is one of the most commonly diagnosed cancers and the leading cause of cancer-related deaths worldwide (Torre et al., 2015; Wood et al., 2015). The non-small cell lung cancer (NSCLC), including squamous cell carcinoma (SCC), adenocarcinoma (AC), and large cell carcinoma (LCC) accounts for approximately 85% of all cases of lung cancer (Hussain et al., 2001). Despite various therapeutic approaches, including chemotherapy, immune checkpoint inhibitors, targeted protein kinase inhibitors et al., the 5-years survival rate of late-stage tumors remains poor with approximately 15% (Novello et al., 2013; Park et al., 2014).

Heat shock protein 90 (HSP90) is a chaperon protein that is highly conserved in various species across the different kingdoms (Chen et al., 2006). There are two major cytosolic isoforms of HSP90AA1, HSP90AB1, and one paralog HSP90B1, also known as GRP94, located in the endoplasmic reticulum in human cells (Chen et al., 2006). HSP90 plays a crucial role in maintaining cellular protein homeostasis by assisting other proteins in folding properly, stabilizing proteins against heat stress, and promoting protein degradation (Schopf et al., 2017).

HSP90 has been shown to regulate a variety of biological processes, including cell growth, differentiation, and survival, particularly in tumor cells (Schopf et al., 2017). Because of the presence of mutant proteins and their rapid proliferation, tumor cells place a greater emphasis on controlling proteostasis (Kamal et al., 2003; Schopf et al., 2017). As a result, HSP90 may be a promising therapeutic target, and several clinical trials are currently underway to assess the efficacy of HSP90 inhibitors alone or in combination with other drugs in the treatment of various types of cancer (Eiseman et al., 2005; Yuno et al., 2018). Geldanamycin (GA) and its analogue 17-allylamino-17-demethoxygeldanamycin (17-AAG or anespimycin) and 17-demethylaminoethylamino-17-demethoxygeldanamycin (17-DMAG or alvespimycin) were discovered as inhibitors of HSP90 (Trepel et al., 2010). They were able to bind to HSP90 at the N-terminal ATP pocket, thus prevents the interaction of the chaperone and the clients (Bucci et al., 2000). When compared with GA or 17-AAG, 17-DMAG exhibits increased water solubility, higher oral bioavailability, longer plasma half-life, less toxicity to normal cells and superior antitumor activity (Kaur et al., 2004; Smith et al., 2005; Trepel et al., 2010). 17-DMAG has been tested in clinical trials and has highly attractive pharmaceutical properties (Egorin et al., 2002). In addition, it is reported that *in vivo* administration of 17-DMAG enhances EphA2+ tumor cell recognition by specific CD8^+^ T cells. And the treatment outcomes were also improved in sarcoma and renal cell carcinoma (Kawabe et al., 2009; Rao et al., 2012). Whether 17-DMAG participated in immune regulation in lung cancer was investigated in our assay.

However, the clinical significance of HSP90 isoforms and the pathways regulated by HSP90 in lung cancer are still unknown. In this study, we compared the transcriptional levels of HSP90 isoforms in cancerous and normal tissue and analyzed the correlation between HSP90 expression and overall survival in multiple datasets. Furthermore, the higher transcription and expression levels of HSP90AA1 and AKT1/ERK pathways were validated in lung cancer patient tissues. In addition, the anti-tumor effect and regulatory pathways of 17-DMAG were tested *in vitro* and *in vivo*.

## Materials and Methods

### Datasets Downloading and Analysis

The transcription data of the lung cancer and normal tissue was downloaded from the GEO database (GSE numbers: GSE10072, GSE32863, GSE19188, and GSE40419) with the “GEOquery” R package (Davis and Meltzer, 2007). The Level 3 mRNA expression data of LUAD was downloaded from the TCGA database. The expression data of normal lungs was downloaded from the GTEX database. The expression values not in log form were log2-transformed. In processing the dataset based on a microarray, the highest probe of the same gene was selected. The correlation between the expression of the indicated genes was analyzed by Spearman’s ranked correlation test. The survival test of the genes was analyzed by the “Survival” R package (Therneau and Grambsch, 2015). Spearman’s rank correlation test was used to determine the correlation of each gene’s expression with HSP90AA1. A positive correlation was defined as rho value greater than >0.3 and *p* < 0.05. And the enrichment of the genes was performed by the “clusterProfiler” (Yu et al., 2012). The protein-protein interaction network was constructed by the String website (STRING, http://www.string-db.org) (Szklarczyk et al., 2019), and the PPI was, analyzed, and visualized by Cytoscape v3.8.2 software (Shannon et al., 2003). The GSEA analysis was performed by GSEA software (Version 4.1.0) (Subramanian et al., 2005), according to the Pearson correlation of HSP90AA1 expression.

### Patients’ Biopsy Specimens

The study was approved by the Ethics Committee in the Medical School of Nanjing University (IRB no.: 20200115003). All patients provided written informed consent. The information details of patients were listed in Supplementary Table S1.

### Cell Lines and Cell Culture

LLC cells were cultured in DMEM (Gibco, Carlsbad, CA) containing 10% fetal bovine serum (FBS, Gibco, Carlsbad, CA) supplemented with penicillin (100 U ml^−1^) and streptomycin (100 µg ml^−1^) in 5% CO2 at 37°C. Cells were obtained from American Type Culture Collection.

Cancer cell lines (A549, Solarbio, China) originating from human lung tumors were cultured in RPMI-1640 medium (Gibco, CA, USA) with 10% fetal bovine serum (FBS; Biological Industries, Israel) and antibiotics (100 μg/ml penicillin-streptomycin, Beyotime, China). The cell cultures were incubated in an environment containing 5% CO_2_ at 37°C.

### Mouse Xenograft Assays

Animal experiments were performed as described previously (Yang et al., 2019). Male Balb/c mice, aged 6–8 weeks were maintained under standard conditions and cared for in accordance with the institutional guidelines for animal care. All the animal experiments were approved by the Institutional Animal Care and Use Committee of Nanjing University.

LLC cells in equal volumes of PBS were subcutaneously injected into the right flanks of mice to establish tumor xenografts (5 × 10^5^ cells per mouse). When the tumor xenografts reached approximately 5 mm in diameter, the mice were randomly divided into treatment and control groups. The treatment group received an intraperitoneal injection of 17-DMAG (MCE, Cat#HY-12024, NJ, USA) (15 mg/kg, daily, dissolved in 1% DMSO +30% PEG300 + 1% Tween 80) every other day. Whereas the control group received a vehicle solvent.

The tumor size was measured with calipers every 2 days. The tumor volume was calculated using the following equation: The tumor volumes were calculated as 1/2 (*length* × *width* ^2^) (Faustino-Rocha et al., 2013). The inhibition rates of 17-DMAG on the tumor were calculated as (average tumor weights of the control group-17-DMAG treated group)/average tumor weights of the control group. At the end of the experiment, the tumors were collected for western blot and histological analysis.

Balb/c Nude (6–8 weeks of age) mice were obtained from the Model Animal Research Center of Nanjing University for this study. The mice were housed under specific pathogen-free conditions at 23°C and given free access to food and water. To prepare A549 tumor xenografts, the left flank of the mice was subcutaneously inoculated with A549 tumor cell suspension (5 × 10^6^ cells/100 μL) (Zhu et al., 2020). Three days after tumor-cell inoculation, the mice were divided into two groups (*n* = 10): Con group (control group, no treatment), 17-DMAG-treated group (15 mg/kg/day), which were administered intraperitoneal injection daily until the end of the experiments. Mice were killed when their minor axis of tumors was longer than 20 mm. All experiments with mice were approved by the ethics committee.

### Cell Viability Assay

Cells were seeded in 96-well plates at 5,000 cells per well in a final culture volume of 100 μL for 24 h before the addition of increasing concentrations of 17-DMAG that were incubated for 24 h. Viable cell number was determined using the Celltiter 96 AQueous Nonradioactive Cell Proliferation Assay (Promega, WI, United States). The value of the background absorbance at 490 nm (A490) of wells not containing cells was subtracted. Percentage of viable cells was calculated as following: A490 of 17-DMAG treated sample/A490 untreated cells × 100. The IC50 was defined as the concentration that gave rise to a 50% viable cell number.

### Cell Apoptosis Assay

For apoptosis detection, the LLC cells or A549 cells were cultured in a six-well plate. Then LLC cells were treated with 17-DMAG or vehicle (DMSO 0.1%) for 24 h; A549 cells were treated with 17-DMAG or vehicle (DMSO 0.1%) for 24 h. Next, the cells were washed once with a complete medium, resuspended in binding buffer, and then stained with Annexin V-fluorescein isothiocyanate and propidium iodide (PI). After 20 min of incubation in the dark at room temperature, stained A549 cells were examined by flow cytometry analysis (BD LSRII SORP, Franklin Lakes, United States). The analysis was repeated three times.

### TUNEL Assay

TUNEL assay was performed by the ApoBrdU DNA Fragmentation Assay Kit (Biovision, San Francisco, CA, United States) following the manufacturer’s instructions. Briefly, the tumorsphere was removed from the implanted region and fixed with 4% paraformaldehyde, and embedded in paraffin. And then, remove the paraffin by immersing the slides in fresh xylene twice. After rehydration, the slides were fixed with 4% paraformaldehyde and washed. Proteinase K was added to remove the remained protein on the slide; then the slides were washed and incubated with a DNA labeling solution. FITC labeled anti-BrdU antibody was added after two washes and then incubated on the slides at RT for 30 min. Then the slides were washed, and PI was adopted to reveal the nuclei of the cells. And the images were captured by the FV10i Laser Scanning Confocal Microscope (Olympus, Center Valley, PA, United States).

### Immunohistochemistry Assay

Immunohistochemistry was carried out according to a standard protocol. Briefly, the tumor spheres were removed from the implanted region and fixed with 4% paraformaldehyde, and embedded in paraffin. After hydrolysis and antigen retrieval, the slides of both tumor-bearing mice and human patients were blocked and washed with PBS. Immunostaining was carried out with a rabbit monoclonal antibody to CD8 at 4°C overnight. Hematoxylin & eosin (H & E) staining was performed for conventional morphological evaluation under a light microscope (Olympus, Tokyo, Japan).

### Western Blot Analysis

The tumor samples were lysed on ice using RIPA buffer containing a cocktail of protease inhibitors and phosphatase inhibitors (Thermo fisher, Cat#78440, CA, United States) with constant shaking for 30 min; cellular debris was pelleted by centrifugation at 10,000 × *g* for 5 min at 4°C, and supernatants were harvested. Protein concentrations were measured by the BCA protein assay kit. Target proteins (30 µg per lane) were separated via SDS-PAGE. Following separation with different concentrations of acrylamide gel (6% for mTOR; 10% for AKT1; 12% for caspase-3 and cleaved caspase-3), the proteins were transferred onto PVDF membranes. The membrane containing the proteins was successively incubated in a blocking buffer (overnight at 4°C), with a primary antibody (37°C for 1 h) and with a secondary antibody (37°C for 1 h). Primary antibodies against HSP-90, CyclinD, pBAD, AKT1, phosphor-AKT1 (Ser473), mTOR, phosphor-mTOR (Ser2448), caspase-3, cleaved caspase-3, ERK and Phospho-p44/42 MAPK (Erk1/2) (Thr202/Tyr204) were purchased from Cell Signaling Technology (Boston, MA, United States). Antibodies against Bcl-xl, Survivin, Mcl-1, and GAPDH were purchased from Abcam (Burlingame, CA, United States). The BCA protein assay kit was purchased from Pierce (Rockford, IL, United States).

### Real-Time Quantitative PCR

Total mRNA was extracted from cultured cells and tumorspheres using the RNeasy Micro Kit (Qiagen, Hilden, Germany), mRNA was reverse transcripted into cDNA with the PrimeScript RT Master Mix (TaKaRa, Otsu, Japan). SYBR green quantitative real-time PCR was performed, using PCR Master Mix (Life Technologies). The relative expression of target genes was determined to beta-actin and was calculated by the ΔΔCt method. Primers: Human AKT1, forward: 5′-GCT​TCT​TTG​CCG​GTA​TCG​TG-3′, reverse: 5′-GGC​CGT​GAA​CTC​CTC​ATC​AA-3′; Human CyclinD1, forward: 5′-GCT​GCG​AAG​TGG​AAA​CCA​TC-3′; reverse: 5′-CCT​CCT​TCT​GCA​CAC​ATT​TGA​A-3′; Human Mcl-1, forward: 5′-GGC​TAA​ACA​CTT​GAA​GAC​CAT​AA-3′, reverse: 5′-GAA​GAA​CTC​CAC​AAA​CCC​ATC-3′; Human Bcl-xl, forward: 5′-AAA​GCG​TAG​ACA​AGG​AGA​TGC-3′, reverse: 5′-TCC​CAT​AGA​GTT​CCA​CAA​AAG​T-3′; Human Survivin, forward: 5′-TTA​CGC​CTG​TAA​TAC​CAG​CAC-3′, reverse: 5′-TCA​CCA​AGG​GTT​AAT​TCT​TCA-3′; Human beta-actin, forward: 5′-GGA​CGA​CAT​GGA​GAA​AAT​CTG-3′, reverse: 5′-GGT​CTC​AAA​CAT​GAT​CTG​GGT-3′.

Mouse AKT1, forward: 5′-ATC​TGA​GTC​CAC​AGC​AAG​GTC-3′, reverse: 5′-GAG​TCT​CTT​CTC​GGT​AGG​CTG-3′; Mouse CyclinD1, forward: 5′-CAA​CTT​CCT​CTC​CTG​CTA​CCG-3′, reverse: 5′-CCT​TGT​TTA​GCC​AGA​GGC​CG-3′; Mouse Mcl1, forward: 5′-AAA​GGC​GGC​TGC​ATA​AGT​C-3′, reverse: 5′-TGG​CGG​TAT​AGG​TCG​TCC​TC-3′; Mouse Bcl-xl, forward: 5′-ACA​TCC​CAG​CTT​CAC​ATA​ACC​C-3′, reverse: 5′-CCA​TCC​CGA​AAG​AGT​TCA​TTC​AC-3′; Mouse Survivin, forward: 5′-GAG​GCT​GGC​TTC​ATC​CAC​TG-3′, reverse: 5′-ATG​CTC​CTC​TAT​CGG​GTT​GTC-3′; Mouse GzmA, forward: 5′-GGG​GGC​CAT​CTC​TTG​CTA​CT-3′, reverse: 5′-TGA​GTG​AGG​AAC​AAC​CGT​GTC-3′; Mouse GzmB, forward: 5′-CAG​GCC​AAT​GGA​ACA​CCT​CT-3′, reverse: 5′-GTG​GAG​AGG​GCA​AAC​TTC​CA-3′; Mouse Ifng, forward: 5′-AGC​AAG​GCG​AAA​AAG​GAT​GC-3′, reverse: 5′-TCA​TTG​AAT​GCT​TGG​CGC​TG-3′; Mouse beta-actin, forward: 5′- GTG​ACG​TTG​ACA​TCC​GTA​AAG​A-3′, reverse: 5′-GCC​GGA​CTC​ATC​GTA​CTC​C-3′.

### Transfection of Small Interfering RNAs (siRNAs)

SiRNA transfection was performed using Lipofectamine RNAimax reagent (Thermo, #13778150, CA, United States), according to the manufacture’s instruction. Briefly, tumor cells were seeded in 6-well plates at 60–80% confluence, 10,000 cells per well in 96-well plates. 25 pmol per well in 6-well plates, and 1 pmol per well in 96-well plates of siRNA was diluted in opt-MEM medium, and mixed with transfection reagent and added in each well. After 48 h of transfection, the efficiency of the transfection and the viability of the cell were evaluated. siRNA sequences: human HSP90AA1 (CUU​CAC​AGA​CUU​GUC​GUU​CUU, scramble: GCG​GCA​CUA​AAA​UUG​UAC​AGG); mouse Hsp90aa1 (CUU​CAC​AGA​UUU​GUC​AUU​CUU, scramble: CAA​AGU​UCU​CAC​UUG​UUU​CUU).

### Statistics and Data Analysis

Each experiment was performed at least three times, and representative data are shown. Data in bar graphs are given as the means ± S.D. Means were checked for statistical difference using the *t*-test and *p*-values less than 0.05 were considered significant (*p < 0.05, **p < 0.01, ***p < 0.001, ****p < 0.0001).

## Results

### HSP90AA1 was Overexpressed and Correlated With Shorter Overall Survival Time in Lung Cancer Patients

**FIGURE 1:**
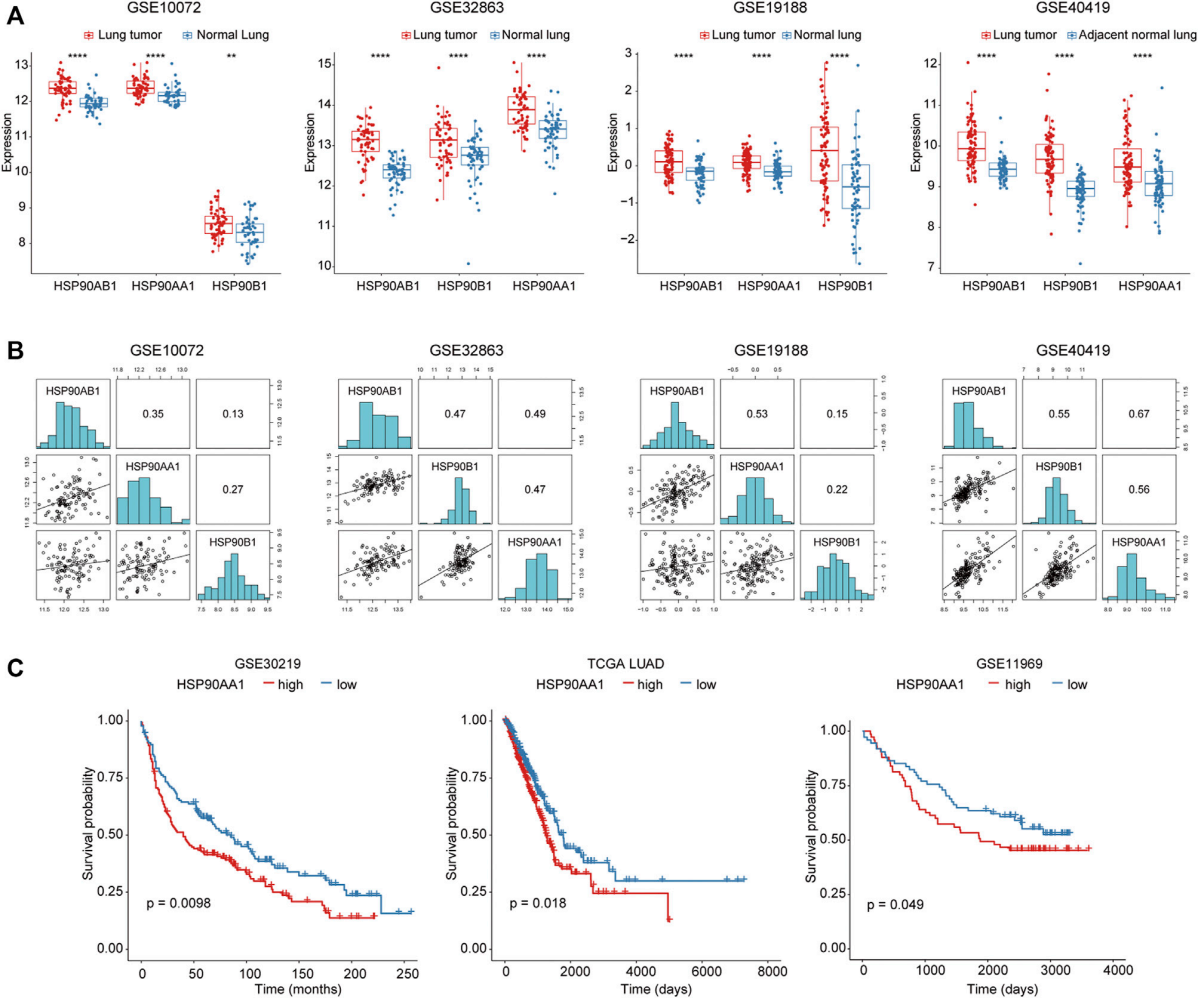
Increased HSP90AA1 is correlated with decreased overall survival time in lung cancer patients. **(A)** HSP90AA1, HSP90AB1, HSP90B1 are all significantly up-regulated in lung cancer tissues in four different high throughput datasets. **p < 0.01, ****p < 0.0001. **(B)** Three HSP90 isoforms (AA1, AB1, B1) are positively correlated with each other. **(C)** HSP90AA1 is significantly correlated with a shortened overall survival time in three datasets.

To investigate the role of HSP90 in lung cancer, the transcription levels of 3 HSP90 isoforms (AA1, AB1, B1) were compared between lung cancer and normal lung tissue in three microarray and one RNA sequencing datasets. As shown in Figure 1A, these three isoforms of HSP90 are all significantly up-regulated in cancer tissues compared with normal lung tissues. Although they were positively correlated with each other (Figure 1B), only the higher expression of HSP90AA1 was significantly correlated with shortened overall survival time in three datasets, including TCGA, in which survival data was available (Figure 1C).

### The Transcription Profile of HSP90AA1 Correlated Genes Was Different Between Lung Cancer and Normal Tissues

**FIGURE 2:**
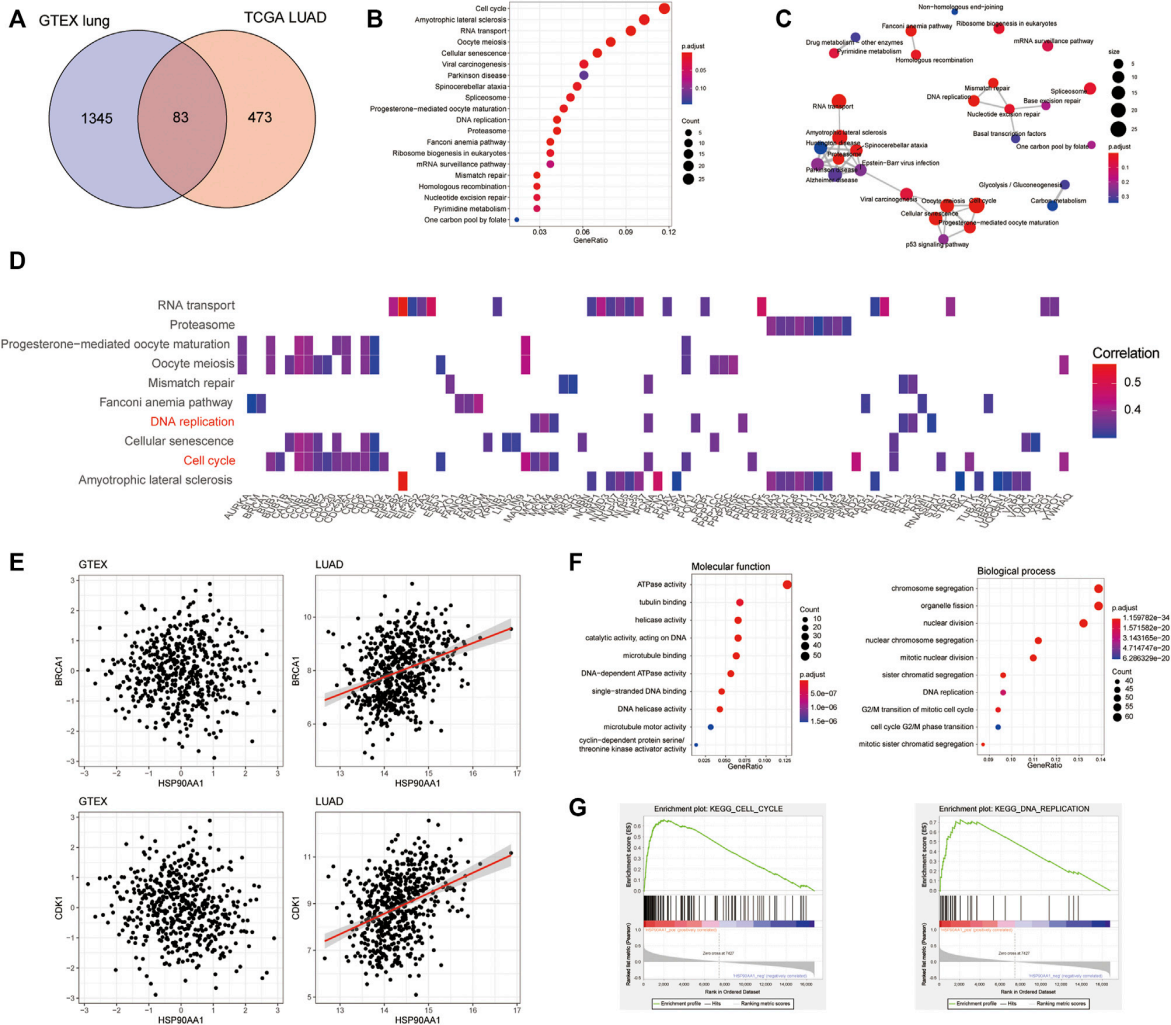
The transcription profile of HSP90AA1 correlated genes was different between lung cancer and normal tissues. **(A)** The Venn plot indicated the overlapping and differences of the genes positively correlated with HSP90AA1 expression in normal (GTEX) and cancerous (TCGA LUAD) lungs. **(B** & **C)** Enrichment and correlations of HSP90 positively correlated genes in KEGG pathways. **(D)** The heatmap of indicated genes enriched in the corresponding pathways and their correlation with HSP90AA1 expression. **(E)** The correlation of BRCA1 and CDK1 with HSP90AA1 in the GTEX and TCGA databases. **(F)** The enrichment of GO terms of molecular function and biological process in HSP90AA1 correlated genes. **(G)** GSEA analysis of HSP90AA1 indicated the possible correlated genes enriched in KEGG pathways of cell cycle and DNA replication.

To further explore the mechanism of HSP90AA1 regulation in cancer development, we tried to compare the differences between the HSP90AA1 correlated genes in normal (GTEX normal lung tissue) and cancer lung (TCGA LUAD) tissues. According to the criteria in the method section (Spearman rho >0.3, *p* < 0.05), 1428 genes in the GTEX database and 556 genes in the TCGA LUAD database were considered as significantly positively correlated with HSP90AA1 expression (Supplementary Figure S1). Interestingly, only 83 genes were shared between normal and lung cancer patients (Figure 2A). There are 473 genes uniquely positively correlated with HSP90AA1 in cancer patients. Next, we enriched the KEGG pathway of these 473 genes in cancer patients. As shown in Figures 2B,C, the KEGG pathways of hsa04110: Cell cycle, hsa03013: RNA transport, hsa03030: DNA replication, and hsa03050: Proteasome, et al., were significantly enriched by these genes. And the heatmap of the enriched genes in the pathways indicated that key genes involving tumor proliferation such as BRCA1, CDK1, and CCNB1 et, al (Figure 2D). The results were further validated in the GTEX database; the correlation between HSP90AA1 and BRCA1 and CDK1 was only observed in cancer patients (Figure 2E). The enrichment of Gene Ontology showed that the molecular function of GO:0008094 DNA-dependent ATPase activity, GO:0003678 DNA helicase activity, and GO:0061575 cyclin-dependent protein serine/threonine kinase activator activity were significantly enriched in these genes. Furthermore, the molecular functions of the biological process, such as GO:0007059 chromosome segregation, GO:0000086 mitotic cell cycle G2/M transition, and GO:0006260 DNA replication, were significantly enriched in these genes. (Figure 2F), implying that HSP90AA1 contributes to tumor cell hyperproliferation.

Besides that, the GSEA assay of the KEGG pathways validated these findings, with the expression pattern of HSP90AA1 positive correlated genes significantly enriched in cell cycle and DNA replication pathways (Figure 2G).

**FIGURE 3:**
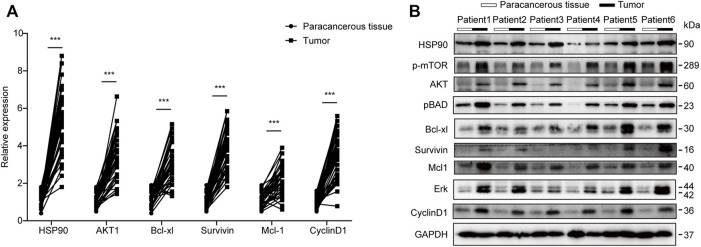
Transcription and western blot validation of AKT1/ERK signaling in lung cancer patients. **(A)** qPCR analysis of HSP90, AKT1, Bcl-xl, Survivin, Mcl1, and CyclinD1 in lung cancer patients’ tumors compared with Para cancerous tissue. Patients number, *N* = 40 The data represents the mean ± standard deviation (SD). ***p < 0.001 **(B)** Western blot analysis of HSP90, AKT1, Bcl-xl, Survivin, Mcl1, and CyclinD1 in lung cancer patient’s tumor compared with Para cancerous tissue. We randomly chose 6 patients and repeated the experiment three times.

To validate this finding, the expression of HSP90AA1 was tested by qPCR and immunoblotting in 40 paired lung cancer and adjacent tissues. HSP90AA1 was consistently up-regulated in lung cancer at both transcriptional and expression levels (Figures 3A,B). The overactivation of AKT and ERK is a common molecular characteristic in lung cancer patients (Riquelme et al., 2016). And in turn, they will regulate a variety of downstream protein substrates, including gGSK3β, mTOR, BAD and CyclinD1, et al., which are critical in the deregulation of apoptosis, proliferation, and cell motility in cancer cells (Liu et al., 2012; Chen et al., 2019). We then found that AKT1/ERK pathways were also significantly activated in these lung cancer tissues, suggesting a possible contribution of HSP90AA1 to their activation.

### HSP90 Inhibitor 17-DMAG Inhibited Lung Cancer Proliferation and Induced Cell Apoptosis

**FIGURE 4:**
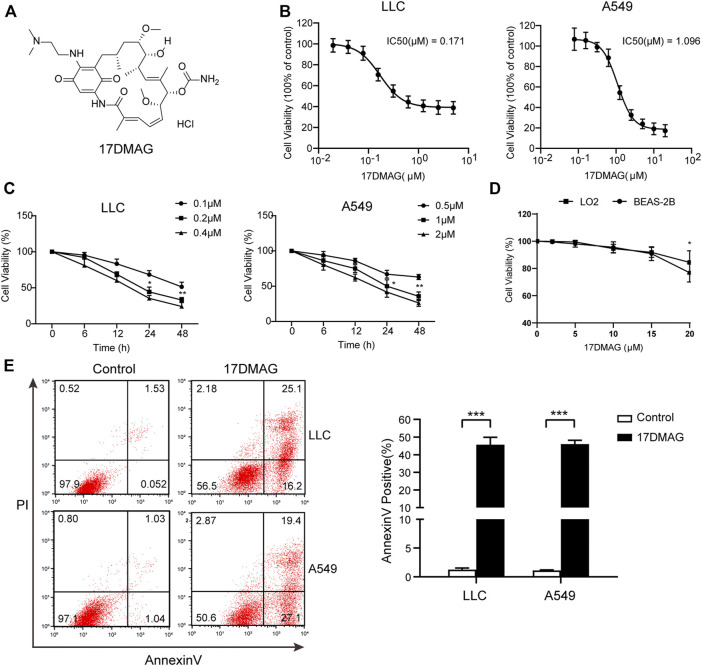
17-DMAG inhibited lung cancer proliferation and induced cell apoptosis. **(A)** Molecular structural formula of 17-DMAG. **(B)** LLC and A549 lung cancer cells were treated with folded diluted 17-DMAG. Cell viability was determined by the MTS method. The IC50 value was determined to evaluate the inhibition of tumor cell growth by 17-DMAG. The value of each cell was the mean value of three independent experiments. **(C)** Time-course analysis of the growth inhibition effect of 17-DMAG in LLC and A549 cells. **(D)** BEAS-2B cells were treated with 17-DMAG at indicated concentrations for 24 h. The cell viability was determined by an MTS assay (*n* = 5). **(E)** LLC and A549 cells were treated with 0.2 or 1 μM 17-DMAG and then stained with PI and AnnexinV-FITC to detect apoptosis by flow cytometry. The columns showed the statistical FACS results of three independent experiments. ***p < 0.001.

17-DMAG, a geldanamycin analog that has been shown to inhibit HSP90 in multiple models, was used to investigate the potential of HSP90 targeting (Ambade et al., 2012; Trepel et al., 2010; Wang Y. L. et al., 2016). Figure 4A depicts the molecular structure of 17-DMAG. In a time-course assay, 17-DMAG significantly inhibited both mouse lung cancer LLC (IC50: 0.171 μM) and human lung cancer A549 (IC50: 1.096 μM) growth in a dose-dependent manner (Figures 4B,C). However, it had no discernible effect on viability in BEAS-2B (a normal human lung epithelial cell line) and LO-2 (a normal human liver cell line) at concentrations more than ten-fold higher than its IC50, indicating its safety (Figure 4D). To see if 17-DMAG inhibited cell proliferation by inducing apoptosis, an AnnexinV-PI cytometry assay was performed. As shown in Figure 4E, 17-DMAG treatment significantly induced massive cell apoptosis in both LLC and A549 cells, implying that its anti-cancer activity is dependent on its pro-apoptotic effect.

### Inhibiting HSP90 Reversed AKT1/ERK Activation in Lung Cancer Cells

**FIGURE 5:**
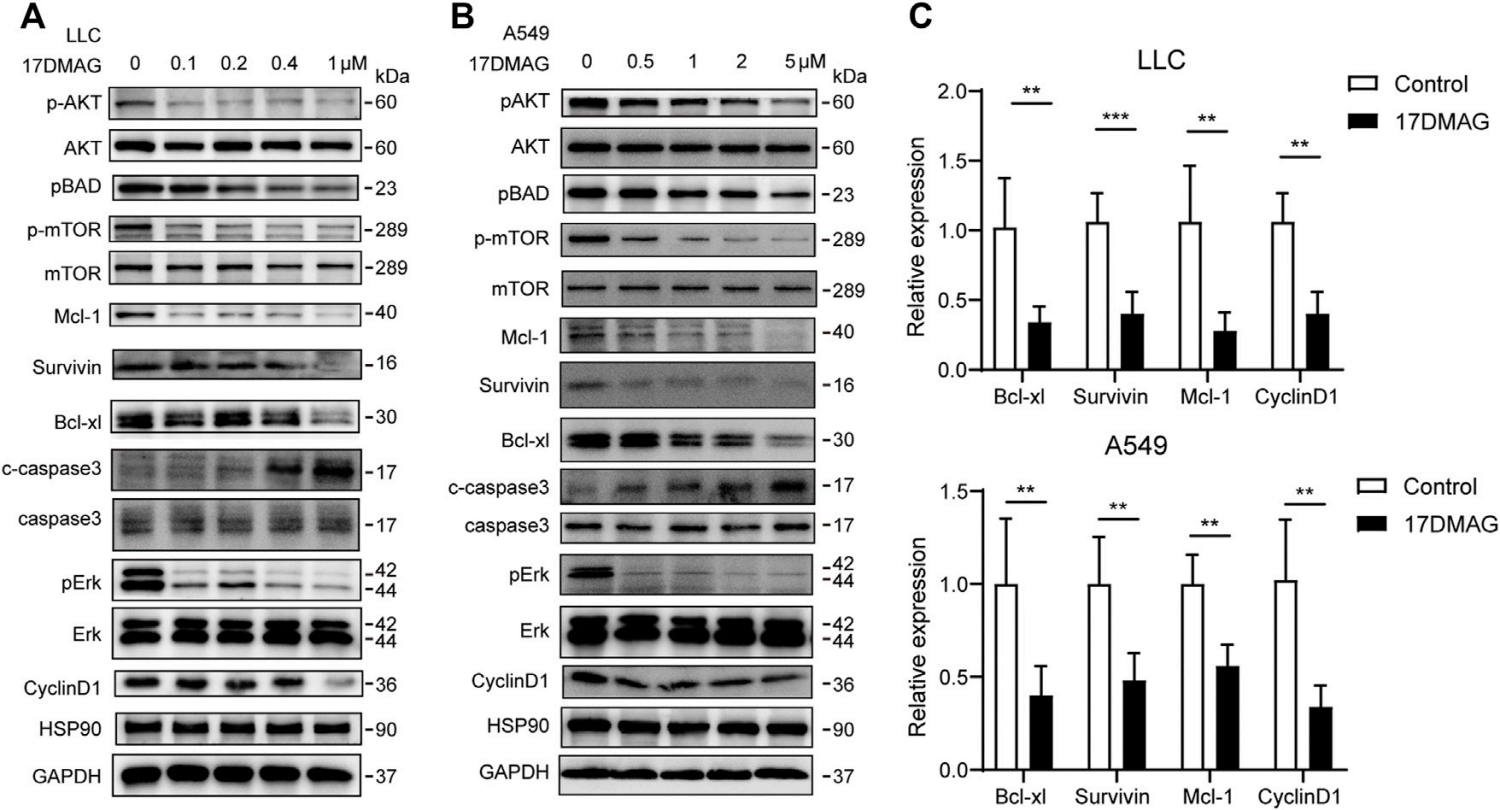
Inhibiting HSP90 reversed AKT1/ERK activation in lung cancer cells. **(A)** LLC cells were treated with indicated concentrations of 17-DMAG and then subjected to western blot for measuring protein levels by indicated antibodies. **(B)** A549 cells were treated with indicated concentrations of 17-DMAG and then subjected to western blot for measuring protein levels by indicated antibodies. **(C)** The total RNA of 17-DMAG-treated LLC cells and A549 cells were extracted, and a qPCR method was adopted to test the transcription level of indicated genes. The result was obtained from three independent experiments. **p < 0.01, ***p < 0.001.

**FIGURE 6:**
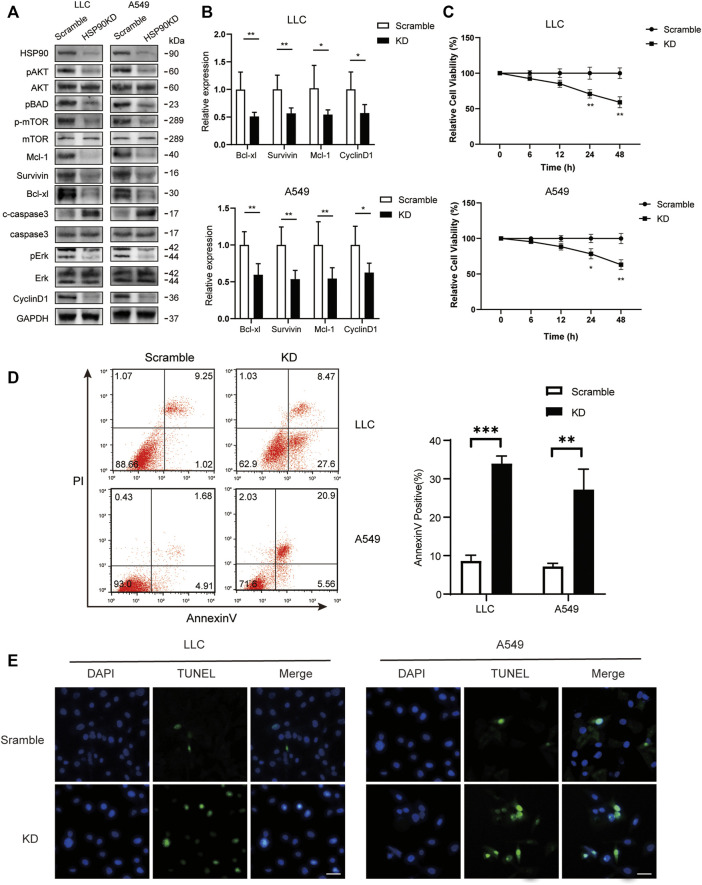
Knocking down of HSP90 inhibited lung cancer proliferation and induced cell apoptosis. LLC and A549 cells were transfected with HSP90 siRNA or control siRNA. **(A)** Western blotting with indicated antibodies was performed. **(B)** The total RNA of the cells was extracted, and a qPCR method was adopted to test the transcription level of indicated genes. **(C)** Time-course analysis of the cell growth detected by MTS assay. *p < 0.05, **p < 0.01. **(D)** The apoptosis level of the cells was evaluated by staining with PI and AnnexinV-FITC antibody and subjected by flow cytometry. The columns showed the statistical FACS results of three independent experiments. **p < 0.01, ***p < 0.001. **(E)** Analysis of apoptosis by TUNEL staining (blue fluorescence, DAPI staining for nuclei; green fluorescence, TUNEL-positive staining) in LLC and A549 cells. Scale bar = 30 μm.

Following that, we attempted to investigate the molecular mechanism of HSP90’s anti-cancer effect in lung cancer. As previously stated, high levels of AKT1/ERK pathways were commonly observed in lung cancers (Li et al., 2012; Wu et al., 2021), which was confirmed in our study (Figure 1D). HSP90 has been shown to regulate the activation of AKT1 and ERK in leukemia and esophageal SCC (Rahmani et al., 2003; Friedman et al., 2013). As a result, we attempted to investigate the possible regulation of HSP90 on AKT1/ERK activation in lung cancer cells. 17-DMAG inhibited AKT1 and ERK phosphorylation in a dose-dependent manner, as shown in Figures 5A,B. The treatment also reduced the phosphorylation of the key pro-apoptotic protein BAD, which was directly phosphorylated by AKT1 (Datta et al., 1997), implying that AKT1 kinase activity was inhibited. In addition, 17-DMAG down-regulated the expressions of several pro-survival proteins, including Mcl-1, Survivin, and Bcl-xl, while up-regulated the levels of cleaved-caspase3. Furthermore, 17-DMAG inhibited the protein and transcriptional levels of CyclinD1, which is important in cell cycle regulation (Figure 5C). We next knocked down HPS90AA1 in these two cell lines to confirm that the pivotal effect HSP90AA1 in lung cancer. In both LLC and A549 cells, knocking down (KD) of HSP90AA1 mimicked the effect of 17-DMAG at protein level and transcriptional levels (Figures 6A,B). It also inhibited the growth of LLC and A549 in a time-course assay (Figure 6C). The AnnexinV-PI cytometry assay and the TUNEL assay demonstrated that HSP90AA1 KD significantly induced cell apoptosis in both LLC and A549 cells, which mimicked the effect of 17-DMAG *in vitro* (Figures 6D,E).

### 17-DMAG Inhibited Lung Cancer Proliferation and Induced Cell Death *in vivo*

**FIGURE 7:**
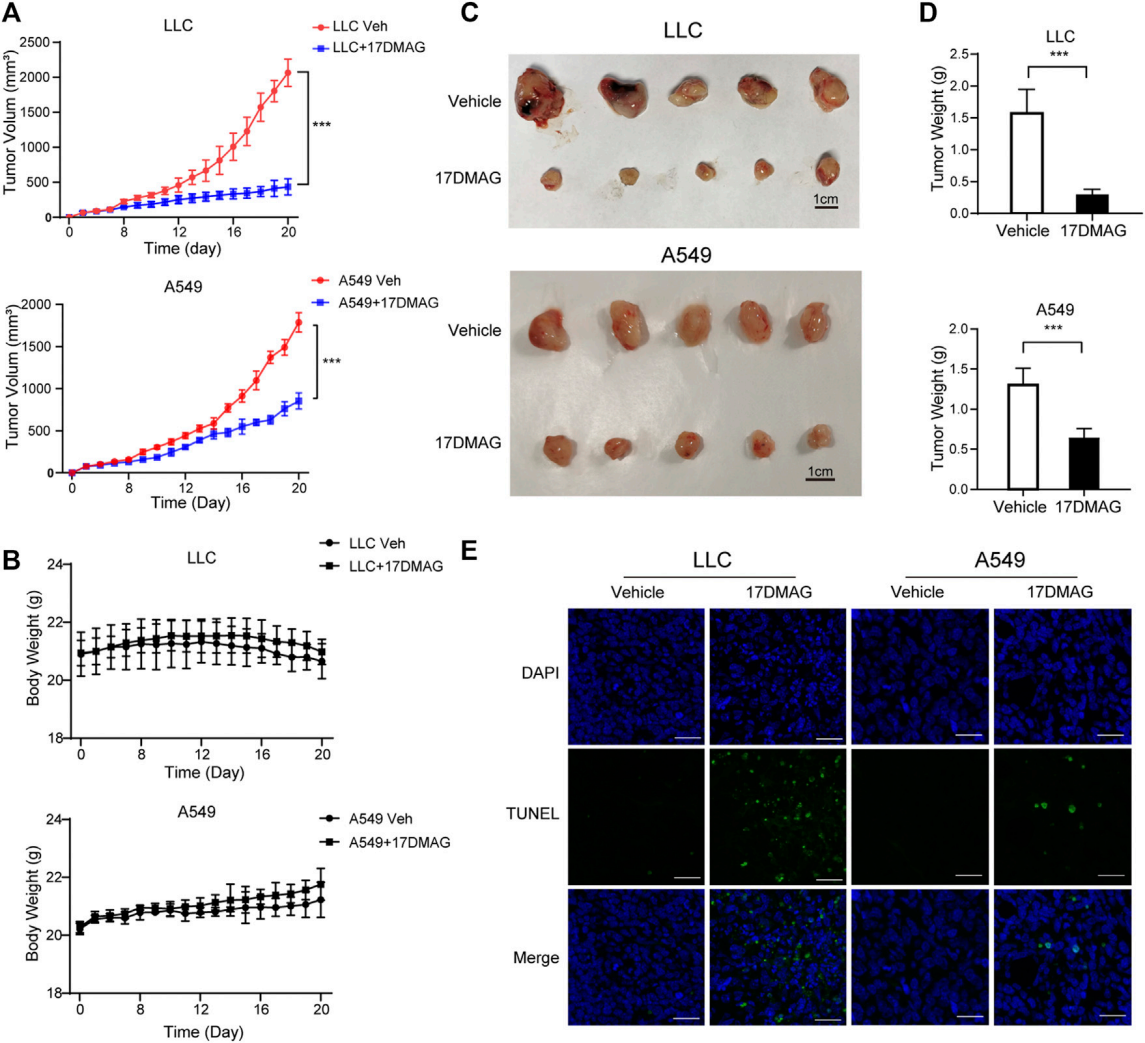
17-DMAG inhibited tumor growth and induced cell death *in vivo*. Male C57/BL6 and Balb/c nude mice (n = 10) were injected with LLC and A549 cells, respectively (*n* = 10). After tumors reached approximately 100 mm^3^, mice were treated with or without 17-DMAG (15 mg/kg/day) intraperitoneally. **(A)** Tumor volumes of LLC and A549 bearing mice were calculated every day. ***p < 0.001. **(B)** The weight of each LLC or A549-bearing mouse was measured every other day before the treatment of 17-DMAG (*n* = 5 per group). **(C)** Tumor mass images of both LLC and A549 model mice at the end of the experiment (*n* = 5 per group). **(D)** LLC and A549 tumors treated with or without 17-DMAG were removed and weighed after the euthanasia of mice. ***p < 0.001. **(E)** Analysis of apoptosis by TUNEL staining (blue fluorescence, DAPI staining for nuclei; green fluorescence, TUNEL-positive staining) in LLC and A549 cell xenograft mouse-derived tissue from control, 17-DMAG–treated animals. Scale bar = 50 μm.

Finally, we investigated 17-DMAG’s anti-cancer activity *in vivo*. Subcutaneous injections of LLC and A549 cells were made into the flanks of C57/BL6 and Balb/c nude mice, respectively. The mice were given either vehicle (PBS) or 17-DMAG (15 mg/kg/day) treatment after the cells formed a 100 mm^3^ sphere. When compared to control groups, both LLC and A549-bearing mice receiving 17-DMAG showed a significant inhibition of tumor growth (Figure 7A). In contrast, the bodyweight of the animals was comparable across groups (Figure 7B), indicating that it was not toxic *in vivo*. The tumors were removed and weighed at the end of the experiment. Tumors in the 17-DMAG group were significantly lighter than those in the control group, and the inhibition rates of 17-DMAG treatment in LLC and A549 bearing mice were 80.9 and 50.7%, respectively (Figures 7C,D). Furthermore, a TUNEL assay revealed that 17-DMAG induced tumor-cell apoptosis *in vivo* (Figure 7E).

**FIGURE 8:**
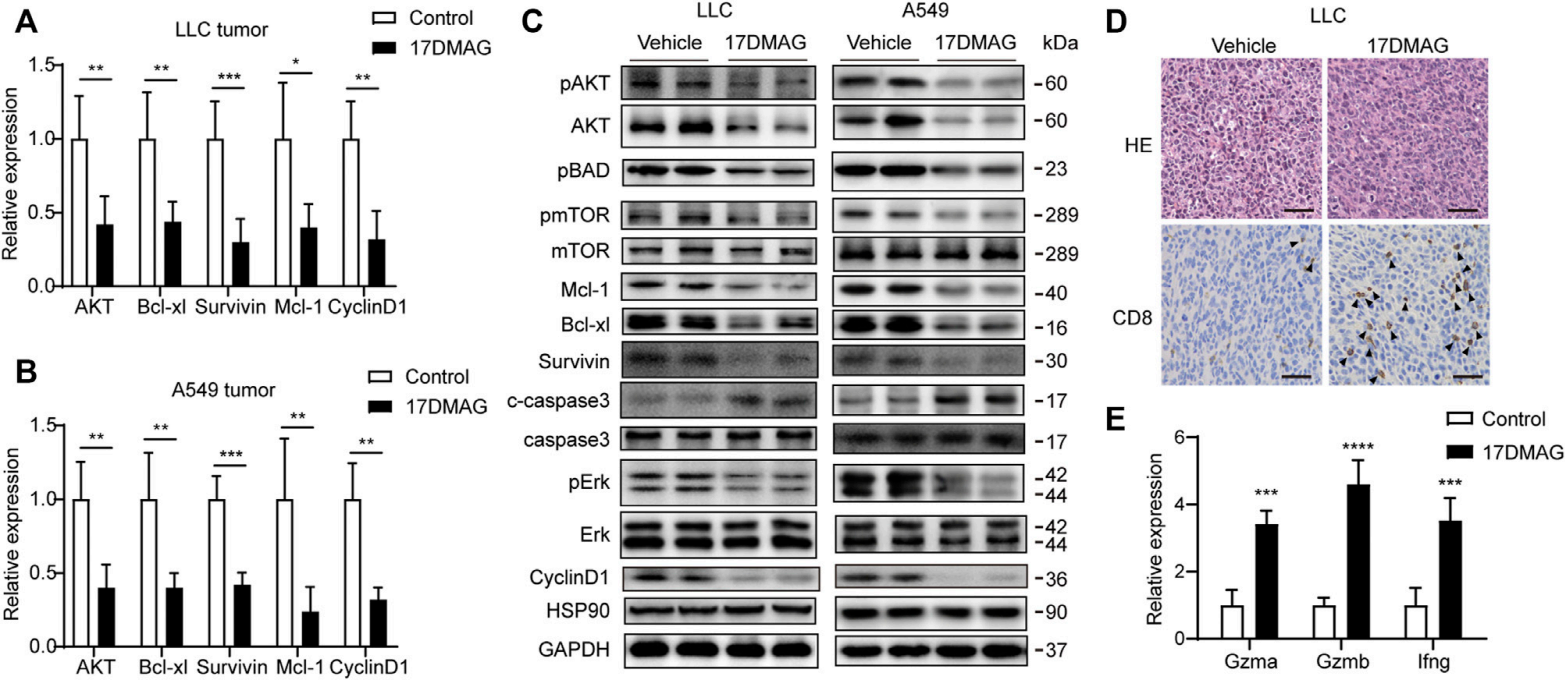
Inhibiting HSP90 reversed AKT1/ERK activation *in vivo*. **(A)** mRNA levels of indicated genes from LLC tumor tissue were tested by qPCR. *p < 0.05, **p < 0.01, ***p < 0.001. **(B)** mRNA levels of indicated genes from A549 tumor tissue were tested by qPCR. **p < 0.01, ***p < 0.001. **(C)** Lysates from LLC and A549 tumor tissue were analyzed by western blotting and probed with indicated antibodies. **(D)** Representative images of LLC tumor sections stained with CD8 antibody. The positive staining of CD8 was marked with arrows. Scale bar = 50 μm. **(E)** Expression of genes related to lymphocyte cytotoxicity. Relative mRNA expression levels were adjusted to the level of beta-actin mRNA for each sample. ***p < 0.001, ****p < 0.0001.

In cancer tissues, the inhibitory effect of 17-DMAG on AKT1/ERK signaling pathways was then investigated. In both tumor models, 17-DMAG inhibited not only AKT1 and ERK phosphorylation but also proliferative markers such as CyclinD1 and induced cleaved Caspase3 expression, indicating a similar effect on HSP90 regulation of the AKT1/ERK pathway *in vitro* (Figures 8A–C). As CD8^+^ T lymphocytes are the major cytotoxic cells that eliminate tumor cells (Prado-Garcia et al., 2012). Immunohistochemical analysis was used to assess the infiltration of CD8^+^ T lymphocytes in the cancer tissues of the immunocompetent mouse C57BL/6 model. As shown in Figure 8D, 17-DMAG treated mice had a significantly higher number of CD8^+^ cytotoxic T cells (marked with an arrow) in tumor tissues compared to vehicle-treated controls. Furthermore, the transcripts of genes related to T-cell–cytotoxic granule components (GzmA and GzmB) and the key anti-tumor cytokine (Interferon-γ) were statistically more abundant in 17-DMAG-treated mice tumors (Figure 8E).

## Discussion

Tumor cells face a more severe proteostasis crisis than normal cells due to the presence of a large number of mutant proteins and a disruption in protein synthesis for rapid proliferation (Sherr, 1996). As a result, HSP90 was important in cancer cells for assisting protein folding and enhancing signaling pathways, making it a potential therapeutic target in cancer research (Wang H. et al., 2016). In human cells, there were two cytoplasmic isoforms of HSP90 proteins named HSP90AA1 and Hsp90AB1, one ER-located isoform of HSP90B1, and one mitochondrial paralog TRAP1. We found that the transcription levels of all these isoforms were higher in cancer tissues than normal tissues (Figure 1A) after a systematic screening of their expression in cancer patients. However, only HSP90AA1 showed a significant correlation with a shorter overall survival time in lung cancer patients, indicating that it plays an important role in tumor progression (Figure 1A).

The serine/threonine kinase AKT (or protein kinase B) controls key cellular processes such as glucose metabolism, cell cycle progression, and apoptosis (Lawlor and Alessi, 2001). Active AKT can contribute to tumorigenesis in a large number of human cancers, including brain, gastric, colon, breast, lung, and prostate carcinomas (Brognard et al., 2001; Scheid and Woodgett, 2001). Hyperactivation of ERK promotes the growth and malignant transformation of lung epithelial cells (Qi et al., 2018; Saad et al., 2019). A constitutively active mutation of KRAS (KrasG12D) and epidermal growth factor receptor (EGFR) can both cause abnormal ERK signaling (Rosell and Karachaliou, 2016; Recondo et al., 2018). Other mechanisms, however, contribute to the dysregulation of this signaling. HSP90 is an ATP-dependent molecular chaperone required for the stability of its "client" oncoproteins, many of which are KRAS effectors, such as members of RAF/ERK and PI3K/AKT/mTOR pathways (Chatterjee et al., 2016). The PI3K/AKT signaling pathway is one of the most frequently activated signaling pathways in different tumors, including lung cancer. The link between HSP90 inhibition and PI3K/AKT/mTOR signaling suppression has been previously reported in a number of solid and hematological malignancies, and our results support these findings (Basso et al., 2002; Ohji et al., 2006; Giulino-Roth et al., 2017). In addition, HSP90 has been shown to be critical for B-RAF or RAF-1–induced MEK activation (Grammatikakis et al., 1999). Concurrent pharmacologic targets of the RAF/MEK/ERK and PI3K/AKT/mTOR pathways have been reported in KRAS-driven cancer models, but clinically this strategy has been limited by additive toxicity (Collisson et al., 2012). In our study, the HSP90AA1 knockdown inhibited the AKT1 and ERK pathways, which were over-activated in tumor tissues.

To explore the clinical translational significance of this observation, we tested the anti-cancer activity of an HSP90 specific inhibitor-17-DMAG in both human and mouse lung cancer cell lines. 17-DMAG is a semi-synthetic geldanamycin derivative, which differs from 17-AAG in position 17 side chain of the ansa ring (Smith et al., 2005). Compared to other HSP90 inhibitors, 17-DMAG was more water-soluble than 17-AAG and had lower toxicity to the liver than geldanamycin, making it a more clinically viable agent.

Our findings show that 17-DMAG has a significant anti-cancer effect with minimal toxicity in LLC or A549-bearing mice. 17-DMAG inhibited cell proliferation and induced cell apoptosis in lung cancer cells by inhibiting the AKT1/ERK pathway, and it was well tolerated in normal cells and mouse models. This study conclusively indicates that targeting HSP90 is a promising therapeutic strategy for patients with lung cancer.

## Data Availability

The raw data supporting the conclusions of this article will be made available by the authors, without undue reservation.
