# Supplementary material for: Targeting HSP90 Inhibits Proliferation and Induces Apoptosis Through AKT1/ERK Pathway in Lung Cancer
Source: Front Pharmacol. 2022 Jan 14;12:724192. doi: 10.3389/fphar.2021.724192 (PMC8795737; doi:10.3389/fphar.2021.724192)
Supplement: Supplementary file 1 [file Table1.pdf]

**Clinicopathological characteristics**	**Male**	**Female**
**Age(years)**	**No. of patients**	
<59	7	5
⩾59	16	12
**Smoking**		
Yes	15	1
No	8	16
**Stage**		
I	0	0
II	11	8
III	12	9
IV	0	0
