# Supplementary figures and images for: Targeting HSP90 Inhibits Proliferation and Induces Apoptosis Through AKT1/ERK Pathway in Lung Cancer
Source: Front Pharmacol. 2022 Jan 14;12:724192. doi: 10.3389/fphar.2021.724192 (PMC8795737; doi:10.3389/fphar.2021.724192)

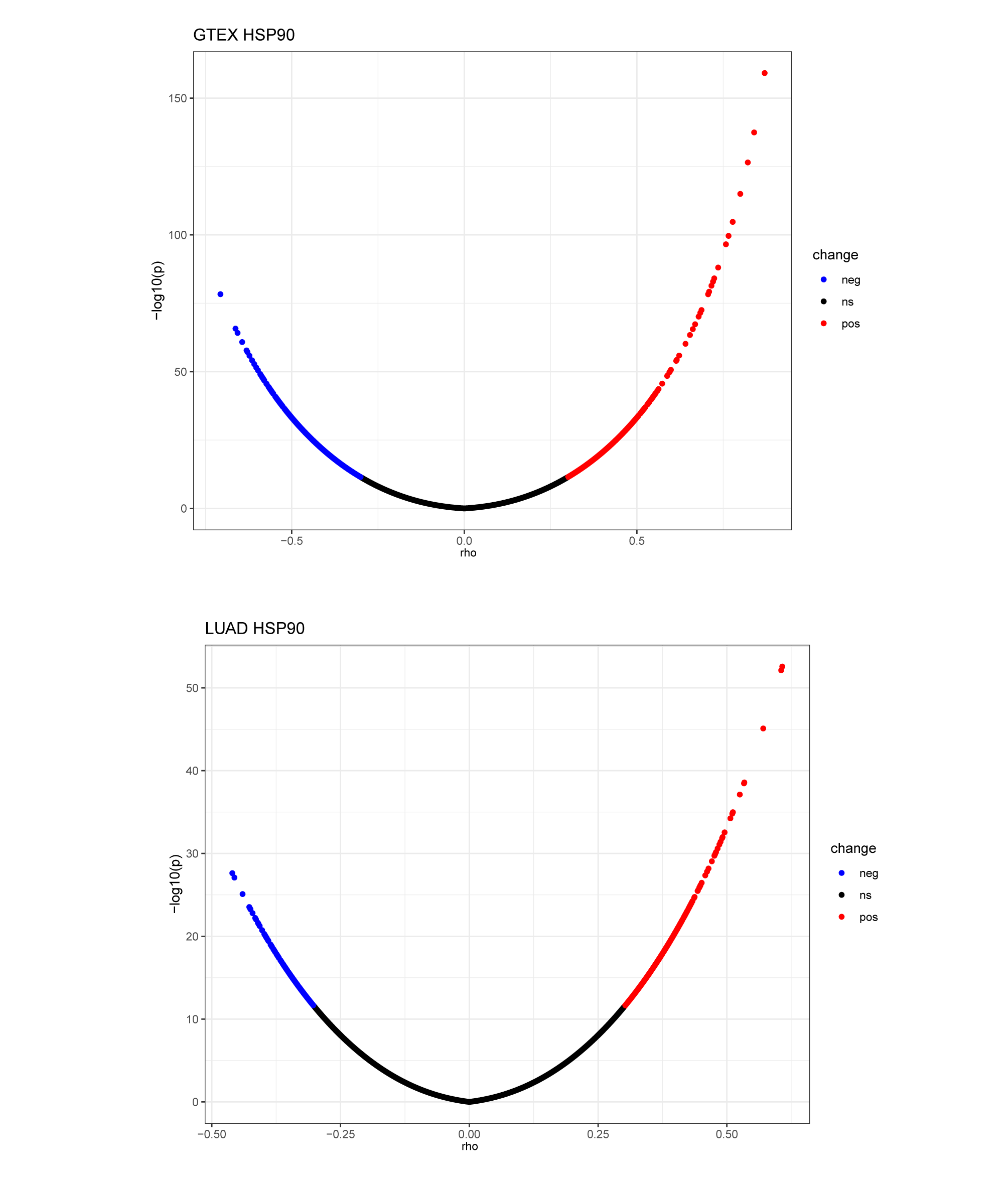

Supplement: Supplementary file 2 [file Image1.TIF]
